# Complete Genome Sequences of 14 Nontuberculous Mycobacteria Type Strains

**DOI:** 10.1128/mra.01214-22

**Published:** 2023-01-18

**Authors:** Yuriko Igarashi, Asami Osugi, Yoshiro Murase, Kinuyo Chikamatsu, Yoshiko Shimomura, Makiko Hosoya, Akio Aono, Yuta Morishige, Hiroyuki Yamada, Akiko Takaki, Satoshi Mitarai

**Affiliations:** a Department of Mycobacterium Reference and Research, Research Institute of Tuberculosis, Japan Anti-Tuberculosis Association, Tokyo, Japan; b Department of Basic Mycobacteriology, Graduate School of Biomedical Sciences, Nagasaki University, Nagasaki, Japan; University of Rochester School of Medicine and Dentistry

## Abstract

Here, we present the complete genome sequences of 14 nontuberculous mycobacteria type strains. The addition of type strain data may provide a concrete basis for further research.

## ANNOUNCEMENT

Nontuberculous mycobacteria (NTM) are generally found in the environment, and disease-causing NTM can affect various organs, especially the lungs. Several new species are registered as NTM every year; however, their whole-genome data are not always available. Here, we report the complete genomes of 14 NTM type strains for which the whole-genome data were not previously registered in the National Center for Biotechnology Information search database (1). The target strains are listed in Table 1 (2–15).

**TABLE 1:** Summary data of 14 NTM type strains

Species name	Strain name	GenBank accession no.	SRA accession no.^*a*^	Long-read sequencing technology	*N*_50_ (bp) (long read)	No. of raw reads (long read)	Coverage (Illumina or PacBio) (×)	No. of contigs	Genome size (bp)	GC content (%)	Total no. of genes^*b*^	Total no. of CDSs^*b*^^,^^*c*^	Growth type	Isolation source^*d*^	Reference(s)
*M. crocinum*	JCM 16369	GCA_022370635.2	SRX17294351 (P)	PacBio	9,821	58,679	46.6	2	5,965,861	67	6,043	5,988	Rapid	Soil	2
Mycobacterium diernhoferi	ATCC 19340	GCA_019456655.1	SRX17145768 (I), SRX17145752 (O)	MinION	2,286	187,432	114.1	1	5,998,503	68	5,716	5,660	Rapid	Soil	3
Mycobacterium goodii	ATCC 700504	GCA_022370755.2	SRX17294352 (P)	PacBio	9,775	77,252	102	1	6,741,281	67	6,421	6,364	Rapid	Calcaneus	4
Mycobacterium heraklionense	JCM 30995	GCA_019645815.1	SRX17145769 (I), SRX17145753 (O)	MinION	10,756	116,682	150.0	1	5,061,737	68	4,681	4,625	Slow	Sputum	5, 6
Mycobacterium holsaticum	JCM 12374	GCA_019645835.1	SRX17145770 (I), SRX17145754 (O)	MinION	7,184	151,826	121.0	1	5,623,714	67	5,359	5,303	Rapid	Sputum	7
*M. lentiflavum*	ATCC 51985	GCA_022374895.2	SRX17294354 (P)	PacBio	9,800	69,110	105	4	5,901,792	66	5,727	5,674	Slow	Disc tissue	8
Mycobacterium malmoense	ATCC 29571	GCA_019645855.1	SRX17145771 (I), SRX17145755 (O)	MinION	7,595	121,181	101.0	1	5,319,859	67	4,926	4,874	Slow	Lung tissue	9
*M. pallens*	JCM 16370	GCA_019456675.1	SRX17145757 (I), SRX17145751 (O)	MinION	4,918	210,816	99.0	3	6,035,548	66	5,956	5,900	Rapid	Soil	2
*M. parakoreense*	DSM 45575	GCA_022370835.1	SRX17294353 (P)	PacBio	9,859	56,061	134	2	3,921,563	70	3,778	3,723	Slow	Sputum	10
Mycobacterium paraterrae	DSM 45127	GCA_022430545.1	SRX17294358 (P)	PacBio	10,417	184,817	318	1	5,522,63	66	5,273	5,216	Slow	Sputum	11
Mycobacterium rufum	JCM 16372	GCA_022374875.1	SRX17294355 (P)	PacBio	9,709	81,388	46.6	2	5,750,471	69	5,804	5,748	Rapid	Soil	2
Mycobacterium senegalense	ATCC 35796	GCA_019645875.1	SRX17145747 (I), SRX17145756 (O)	MinION	6,751	153,355	100.0	1	6,086,722	67	5,788	5,730	Rapid	Bovine lymph node	12, 13
M. ulcerans	ATCC 19423	GCA_022374915.1	SRX17294356 (P)	PacBio	17,027	58,519	46.6	1	5,624,909	65	5,140	5,088	Slow	Leg ulcer	14
Mycobacterium virginiense	DSM 100883	GCA_022374935.2	SRX17294357 (P)	PacBio	10,394	135,308	273	1	4,980,168	67	4,713	4,658	Slow	Flexor tendon	15

a I, MiSeq (Illumina); O, MinION (Oxford Nanopore Technologies); P, PacBio (Pacific Biosciences).

b Excluding plasmid.

c CDSs, coding DNA sequences.

d All isolation sources are of human origin except for samples isolated from bovine and soil.

Each type strain was shared from American Type Culture Collection, Japan Collection of Microorganisms, and German Collection of Microorganisms and Cell Cultures. The strains were grown in Middlebrook 7H10 agar medium (Becton Dickinson, USA) and incubated for 1 to 3 weeks at 30°C or 37°C according to the optimal conditions for each species. DNA was extracted using the conventional phenol-chloroform method after enzymatic (100 μg/mL proteinase K with 2% SDS) treatment or by bead beating (0.2-mm diameter zirconia beads; 3 min) (16). The extracted DNA sequences were analyzed using the MinION (Oxford Nanopore Technologies [ONT]) or PacBio Sequel II (Pacific Biosciences, USA) (Table 1) long-read sequencing technologies. For MinION sequencing, libraries were generated using the SQK-RBK004 kit (ONT) and sequenced using the FLO-MIN106D R9.4 GridION flow cell (ONT). Base calling was performed using Guppy v4.2.2 (ONT). For PacBio Sequel II sequencing, DNA was fragmented using g-TUBE (Covaris, USA), sequencing libraries were prepared by using the PacBio SMRTbell express template prep kit 2.0 (Pacific Biosciences), and libraries between 8 and 20 kb were selected using BluePippin (Sage Science, USA). Circular Consensus Sequencing (CCS) was performed using the PacBio sequel II sequencing kit v2.0. High-fidelity (HiFi) reads were generated from the obtained sequences using the ccs package v6.4.0 in PacBio tools distributed via bioconda (17). Only PacBio Sequel II sequencing was commissioned by the Kazusa DNA Laboratory (Kisarazu, Japan). Short-read sequencing was also performed by MiSeq (Illumina, USA) for the samples analyzed by MinION. For short reads, libraries were generated with the QIAseq FX DNA library kit (Qiagen, USA), and 300-bp paired-end sequencing reactions were performed using the MiSeq reagent kit v3 (600 Cycles) (Illumina). All the procedures associated with the sequencing were performed following the manufacturer’s instructions. Sequence quality was assessed using NanoPlot (18), longQC (19), and FastQC (20) for the MinION reads, HiFi reads, and short reads, respectively. Both types of long reads were assembled into circular contigs using Flye v2.8.3-b1705 (21). For MinION reads, each contig was polished twice using short reads and MinION reads using Pilon v1.23 (22) and racon v1.4.22 (23), respectively. All generated chromosomes were rotated using the circulator v1.5.5 (24) to start at *dnaA* whose sequence was predicted by prokka v1.14.6 (25). The genome was annotated using the NCBI Prokaryotic Genome Annotation Pipeline (26). Default software parameters were used unless otherwise noted.

A summary of the sequence results is shown in Table 1. Five strains––Mycobacterium crocinum, Mycobacterium lentiflavum, Mycobacterium pallens, Mycobacterium parakoreense, and Mycobacterium rufum––had 2 to 4 closed circular contigs, indicating the presence of plasmids.

### Data availability.

The genome sequences and BioSample numbers are available at GenBank (BioProject accession number PRJNA747101), and all accession numbers can be found in Table 1.
